# Facile Strategy of Improving Interfacial Strength of Silicone Resin Composites Through Self-Polymerized Polydopamine Followed via the Sol-Gel Growing of Silica Nanoparticles onto Carbon Fiber

**DOI:** 10.3390/polym11101639

**Published:** 2019-10-10

**Authors:** Yawen Zheng, Xiaoyun Wang, Guangshun Wu

**Affiliations:** School of Chemistry and Materials Science, Ludong University, Yantai 264025, China; zheng1ya2wen3@163.com (Y.Z.); wxyangao@163.com (X.W.)

**Keywords:** carbon fibers, surface modification, silica nanoparticles, interface, polymer-matrix composites

## Abstract

In the present research, to enhance interfacial wettability and adhesion between carbon fibers (CFs) and matrix resin, hydrophilic silica nanoparticles (SiO_2_) were utilized to graft the surface of CFs. Polydopamine (PDA) as a “bio-glue” was architecturally built between SiO_2_ and CFs to obtain a strong adhesion strength and homogenous SiO_2_ distribution onto the surface of CFs. The facile modification strategy was designed by self-polymerization of dopamine followed by the hydrolysis of tetraethoxysilane (TEOS) onto carbon fibers. Surface microstructures and interfacial properties of CFs, before and after modification, were systematically investigated. The tight and homogeneous coverage of SiO_2_ layers onto the CF surface, with the assistance of a PDA layer by self-polymerization of dopamine, significantly enhanced fiber surface roughness and wettability, resulting in an obvious improvement of mechanical interlocking and interfacial interactions between CFs and matrix resin. The interlaminar shear strength (ILSS) and the interfacial shear strength (IFSS) of CF/PDA/SiO_2_ reinforced composites exhibited 57.28% and 41.84% enhancements compared with those of untreated composites. In addition, impact strength and the hydrothermal aging resistance of the resulting composites showed great improvements after modification. The possible reinforcing mechanisms during the modification process have been discussed. This novel strategy of developed SiO_2_-modified CFs has interesting potential for interfacial improvements for advanced polymer composites.

## 1. Introduction

Carbon fiber (CF) reinforced polymer composites with strong strength, light weight, and environmental stability have been widely used as structural materials in aerospace, automotive, and defense industries [1,2,3,4,5]. However, the lack of polar groups and the smooth graphitic fiber surface result in a poor-quality interface between CFs and matrix resin [6,7], limiting wide-ranging application of the composites. The mechanical properties of composites mainly depend on the interface between fibers and the matrix [8,9], and interfacial properties are strongly dependent on time, temperature, and fiber orientation. Zhandarov et al. [10] reported a valuable strategy for estimating local fiber/matrix interfacial strength parameters from micro-bond test data. Almeida Jr. et al. [11] studied the effects of fiber orientation on interlaminar and in-plane shear properties of glass fiber/epoxy composites using four different shear test methods; they also investigated the interfacial and creep characteristics of carbon fiber-reinforced epoxy laminates with different fiber orientations [12]. There are several mechanisms for fiber-matrix bonding, like chemical bonding, mechanical interlocking, physical adsorption, and interfacial wettability, which can affect interfacial adhesion between CFs and the matrix. Therefore, to obtain an optimized engineered interface for advanced polymer composites, many active works have been proposed, such as physical sizing or coating [13,14], chemical modification [15,16], oxidation or plasma treatment [17,18], and high energy irradiation [19].

In recent studies, CFs modified using nanoscale materials for notably improving interfacial wettability and adhesion with matrix resin have attracted increasing attention and have become more popular [20,21,22,23]. Noteworthy, nano-sized SiO_2_ with the unique spherical structure and high physicochemical properties has been smartly utilized for surface modification of CFs with the aim of constructing multi-scale and hierarchical structures [24,25,26]. Typically, the uniform dispersion and tight binding of nano-sized SiO_2_ onto CF surfaces has been considered as a vital factor for obtaining a high interfacial strength as well as their wide application. Therefore, several studies have been reported that firmly introduce SiO_2_ onto the fiber surface by chemical grafting to change fiber surface structures and composite interfacial properties [27,28]. However, these procedures either involve strong acids, toxic polar organic reagents, high energy consumption, or multistep manipulation, which easily form many defects and weak spots and thus damage a fiber′s intrinsic tensile strength. In addition, they cause energy consumption and chemical pollution, which inevitably impede environmental and sustainable development. Hence, it is imperative to explore a powerful and facile approach to disperse SiO_2_ well onto the surface of CFs and obtain a strong interfacial adhesion between CFs and SiO_2_ for advanced polymer composites.

Recently, dopamine (DA) with abundant catechol groups and ethylamino groups could self-polymerize under mild conditions and form polydopamine (PDA) layer on almost all types of substrates [29,30]. Moreover, based on the secondary reactivity of PDA, PDA could provide a versatile and robust platform for further grafting. Zhang et al. [31] grafted polyamide (PA) thin-film composite (TFC) membranes using TiO_2_ nanoparticles with the help of a PDA coating for multifunctional applications, and found that the PDA can readily bind TiO_2_ to membranes. Based on the nature and ability of PDA, modifying a CF′s surface with dopamine chemistry to enhance the interfacial strength of their composites has been employed [32,33]. However, studies on SiO_2_ grown on the surface of CFs pre-modified by a PDA coating and investigating their interfacial reinforcing mechanisms for advanced composites have not been reported.

In the present study, we reported a mild and effective method to construct a multifunctional SiO_2_ layer on a fiber surface with the aid of a PDA coating to simultaneously improve interfacial strength and environmental stability of composites. Biomimic PDA, with providing enough binding force for further SiO_2_ grafting, was firstly coated onto the CFs surface by self-polymerization. Then, SiO_2_ nanoparticles were uniformly deposited onto the surface of CFs via the hydrolysis of tetraethoxysilane (TEOS). During the facile procedure, no harsh conditions and toxic solvents were needed. Modified CFs have been physico-chemically characterized in more detail using scanning electron microscopy (SEM), X-ray photoelectron spectroscopy (XPS), and dynamic contact angle analysis (DCA). The interfacial properties (ILSS and IFSS), impact strength and hydrothermal aging resistance of untreated and treated composites have been thoroughly studied. In addition, interfacial reinforcing mechanisms have been also examined.

## 2. Materials and Methods

### 2.1. Materials

Carbon fibers with a monofilament diameter of 7 μm were purchased from Toray Industries, Inc. (Tokyo, Japan) The fiber sizing agents were removed by Soxhlet extraction with acetone before using, donated as untreated CF. Tris(hydroxyl-methyl)aminomethane (Tris) and dopamine hydrochloride were received from Aladdin (Shanghai, China) and Sigma-Aldrich (Shanghai, China), respectively. Methylphenylsilicone resin (MPSR) was provided by ShangHai Chemicals Co. (Shanghai, China) TEOS, ammonia, acetone, hydrochloric acid (HCl), and ethanol were purchased from Tianjin Bodi Organic Chemicals Co. Ltd. (Tianjin, China).

### 2.2. Preparation of PDA/SiO_2_ Coated CFs (CF/PDA/SiO_2_)

**Figure 1:**
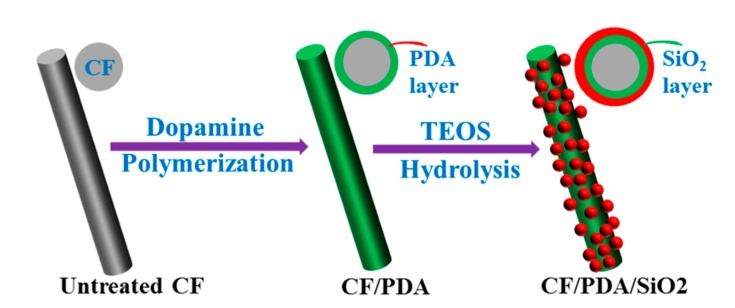
Schematic of binding SiO_2_ coating onto the modified fiber surface via self-polymerized polydopamine.

Modifying the CF surface by self-polymerization of dopamine followed by hydrolysis of TEOS was carried out using the following procedures. Untreated CF was directly immersed in an aqueous dopamine solution, which was prepared by dissolving dopamine in Tris-HCl solution (pH = 8.5), and allowed to react at 25 °C for 6 h to obtain polydopamine-coated CFs by self-polymerization of dopamine (denoted as CF/PDA). After being washed with deionized water to thoroughly remove unreacted dopamine, CF/PDA was immersed into an alcohol–water mixed solution of 82 mL ethanol and 3.4 mL water by stirring for 10 min. Afterwards, 5.6 mL ammonia solution was used as a catalyst and 0.05 mol/L TEOS were quickly added into the solution and allowed to react at 25 °C for 15 h. After the reaction, the modified fibers, which were donated as CF/PDA/SiO_2_, were washed repeatedly with deionized water and dried. Figure 1 shows a schematic outlining of the PDA coating, followed by the growth of SiO_2_ onto the fiber surface by TEOS hydrolysis.

### 2.3. Preparation of CF/MPSR Composites

MPSR composites reinforced by untreated and modified CFs were prepared by using the compression molding method according to our former work [4]. First, a metal frame wrapped around the CFs dissolved in MPSR solution to make the resin fully saturate into CFs, and then the unidirectional fiber prepreg was obtained. Next, the prepreg was placed into the given mold and cured using a hot press machine under the controlled conditions described in a previous paper [16]. The fiber contents in composite laminates were nearly in the range of 60% to 70%, and the laminate’s dimensions of about 2 mm × 20 mm × 6 mm were used for property testing.

### 2.4. Characterization Techniques

X-ray photoelectron spectroscopy (XPS, ESCALAB 220i-XL, Thermo VG Scientific, UK) was performed to study the surface chemical composition and functional groups of CFs surface with and without modifications. XPS measurement was carried out using a monochromatic Al Ka source of 1486.6 eV at a base pressure of 2 × 10^−9^ mbar. The XPS peak version 4.1 program was used for data analysis.

Scanning electron microscopy (SEM, Quanta 200FEG, Hitachi Instrument Inc., Japan) was utilized to observe surface morphologies of CFs before and after grafting and the fractured microstructures of different composites. All fiber and composite samples needed to be sputtered with gold before SEM analysis to increase sample conductivity to obtain stable and clear images.

A dynamic contact angle meter (DCAT21, Data Physics Instruments, Stuttgart, Germany) was used to examine dynamic contact angles for evaluating fiber wettability and surface energies. It was recorded by immersing CFs into two testing solutions (deionized water and diiodomethane), and followed by the calculation of fiber surface free energy, and its dispersive and polar components using the obtained contact angles and the known surface energies of testing liquids.

The ILSS values of CF/MPSR composites were tested on a universal testing machine (WD-1, Changchun, China) using a three-point short-beam bending testing mode. 

Single filament pull-out tests (FA620, Tokyo, Japan) were used to examine interfacial adhesion between CFs and MPSR by pulling a single fiber out from the cured resin droplets. A carbon fiber monofilament was fixed on a metal holder with adhesive tape. Microdroplets were prepared by dropping small resin droplets with a pin and followed by the controlled curing procedure. 

Impact toughness of different composites were studied via a drop weight impact test system (9250HV, Instron, Boston, Massachusetts, USA). The impact span, drop weight of the wedge-shaped impactor, and velocity were 40 mm, 3 kg and 1 m s^−1^, respectively.

To investigate effects of surface modification onto composites hydrothermal aging resistance, interfacial strength of composites which immersed in the boiling water for 48 h were characterized, and traced the changes in ILSS values compared to ILSS results without aging.

## 3. Results

### 3.1. Surface Composition and Microstructure of CFs

**Figure 2:**
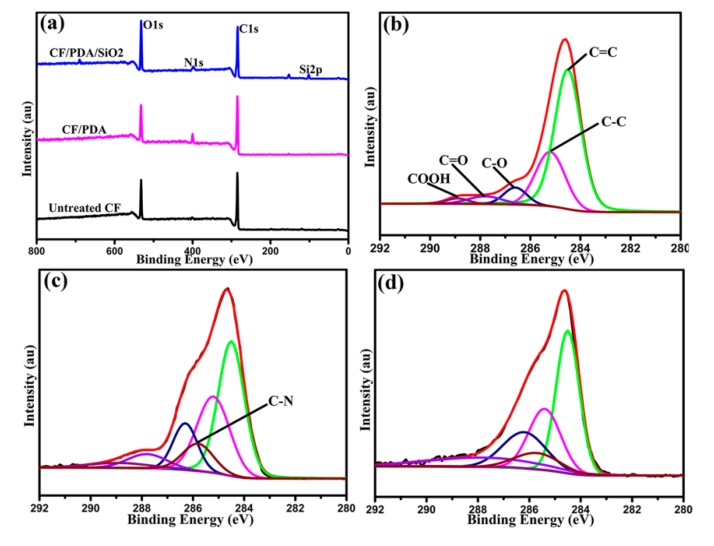
(**a**) Wide-scan survey XPS spectra of carbon fibers; XPS spectra of C1s peaks: (**b**) untreated CF, (**c**) CF/PDA, and (**d**) CF/PDA/SiO_2_.

Surface elements and groups of different CFs were analyzed using XPS. Figure 2 shows the wide-scan and high-resolution XPS spectra of untreated and modified CFs. According to the wide spectra results (Figure 2a), there are C1s and O1s peaks in the wide spectra of untreated CF. After the introduced PDA coating, CF/PDA displays the new peak of N1s, indicating the successful deposition of the PDA layer onto the untreated CF surface. After the hydrolysis of TEOS onto CF/PDA surface, the appearance of S2p peak implies that SiO_2_ nanoparticles have been bound onto the surface of CF/PDA. The binding characterizations for different CFs have also been determined by the high-resolution XPS C1s spectra, as shown in Figure 2b–d. For untreated CF (Figure 2b), there were five peaks (C=C (284.5 eV), C–C (285.2 eV), C–O (286.6 eV), C=O (287.8 eV), and COOH (288.9 eV) onto the fiber XPS spectrum [34]. Compared to untreated CF, the C–N peaks (285.7 eV) appeared in CF/PDA, and the contents of C–O and C=O peaks increased obviously, owing to the formed PDA coating by the self-polymerization of DA. CF/PDA/SiO_2_ presents the decrease of C–N contents and the increase of C–O contents attributed to the sol-gel growing of SiO_2_ nanoparticles onto CFs.

**Figure 3:**
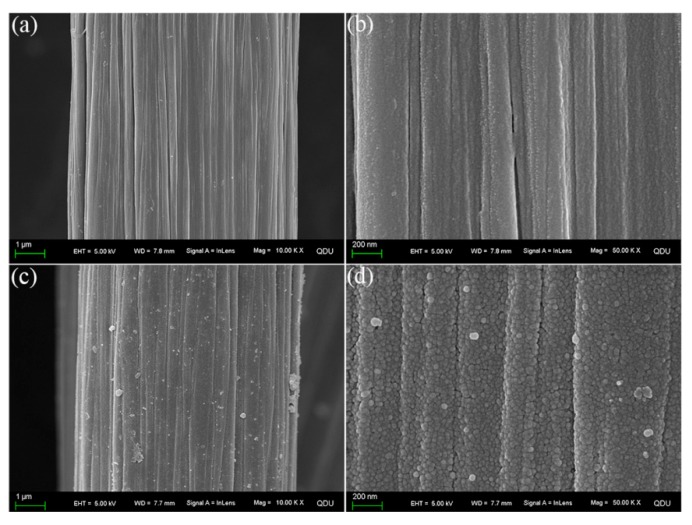
SEM images of different CFs surfaces: (**a**,**b**) CF/PDA and (**c**,**d**) CF/PDA/SiO_2_.

Surface microstructures of untreated and modified CFs are presented in Figure 3. As is known, the surface of untreated CF is typically smooth and neat [7]. After the self-polymerization of PDA, the surface of CF/PDA (Figure 3a,b) becomes rougher compared to that of untreated CF, due to the coverage of PDA layer onto the fiber surface. The formed PDA acts as, not only the active platform for SiO_2_ grafting, but also as a shielding layer for hydrothermal aging. This surface morphology is analogous to that of fibers grafted with PDA coating [33]. As for CF/PDA/SiO_2_ (Figure 3c,d), many nodule-like structures are observed on the fiber surface owing to the hydrolysis of TEOS in situ and the introduction of SiO_2_ nanoparticles. The PDA/SiO_2_ layer not only augments the surface roughness and improves the capacity of mechanical interlocking, but also creates many curing reactive areas to introduce chemical bonding at the interface between CFs and matrix resin, with the aim of achieving outstanding interfacial properties.

### 3.2. Surface Wettability Analysis of CFs

High fiber surface energy is critical to enhance the specific activity and wettability with matrix resin, and results in a good quality of fiber–matrix interface. Hence, dynamic contact angles and surface energy have been carried out to evaluate the relative wettability of different CFs with matrix resin. The dynamic contact angles and surface energy of untreated and modified CFs are summarized in Table 1. Based on the non-ideal graphitic basal planes on the surface of untreated CF, *θ* of untreated CF in water (*θ*_water_) and in diiodomethane (*θ*_diiodomethane_) are 78.50° and 58.91°, respectively. It is well known that the smaller contact angle indicates the better wettability of CFs. Hence, Surface energy of untreated CF is only 35.86 mN·m^−1^, with the dispersion component of 29.20 mN·m^−1^ and the polar component of 6.66 mN·m^−1^. After being introduced by PDA coating, *θ*_water_ of CF/PDA decreases from 78.50° to 51.36°, whereas *θ*_diiodomethane_ decreases from 58.91° to 49.72°; the surface energy of CF/PDA increases up to 54.17 mN·m^−1^. The phenomenon also confirms that the PDA layer containing hydrophilic groups has been successfully deposited onto the surface of CFs. After SiO_2_ grafting, *θ*_water_ and *θ*_diiodomethane_ decrease sharply to 44.32° and 43.26°, the main contributors being the improved polar groups and surface roughness. Compared to those of untreated CF and CF/PDA, the surface energies of CF/PDA/SiO_2_ increases to 60.18 mN·m^−1^ by 67.82% and 11.10%, respectively. As a result, the obvious increase of surface energy can facilitate the resin impregnation and enhance curing reactivity and wettability between CFs and MPSR aiming to improve composites interfacial strength.

### 3.3. Interfacial Property Testing of Composites

**Figure 4:**
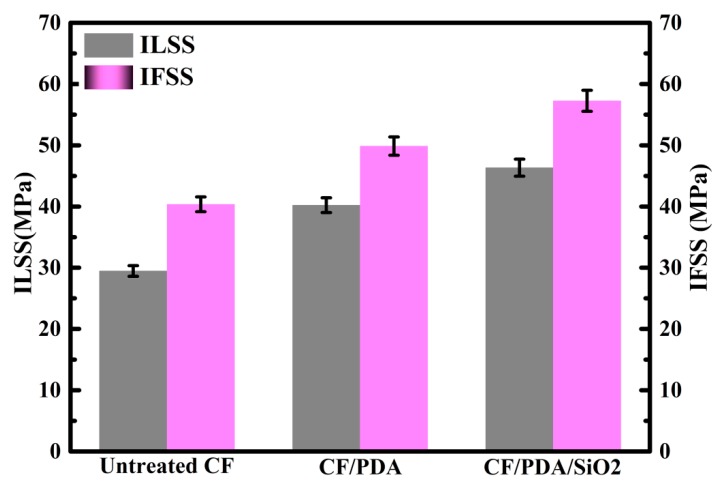
ILSS and IFSS results of composites.

A good quality of fiber-matrix interface is a key factor to increase the integrative mechanical properties of resultant composites. The ILSS and IFSS results of MPSR composites reinforced by different CFs are presented in Figure 4. ILSS and IFSS values of untreated CF composites are only 29.47 and 40.37 MPa, respectively. As for CF/PDA composites, they reach 40.23 MPa with a 36.51% amplification for ILSS and 49.87 MPa improved by 23.53% for IFSS as compared to those of untreated CF composites. This may be due to the improved wettability and interfacial interaction between CFs and matrix resin by introducing many polar groups onto the fiber surface after PDA grafting. As for CF/PDA/SiO2 composites, the recorded ILSS value is 46.35 MPa (57.28% amplification to that of untreated composites), whereas the recorded IFSS value is 57.26 MPa (42.19% amplification to that of untreated composites). Compared to CF/PDA composites, PDA/SiO_2_ deposition combines more active groups and bigger surface roughness, and exhibit a synergistic effect, leading to higher amplifications in ILSS and IFSS evaluation of the resulting composites. 

In general, the reinforcing mechanisms of interfacial strength may be due to the introduced mechanical interlocking, the increased surface wettability, as well as the formed chemical bonding at the fiber–matrix interface. After being introduced to PDA/SiO_2_ layers, polar hydroxy groups can enhance fiber surface free energy, and make the surface of CFs easier to be wetted with matrix resin, which maximizes the degree of molecular contact. Moreover, the polar groups as the curing agents can react with matrix resin to form chemical bonding at the interface. In addition, PDA/SiO_2_ deposition layers onto the fiber surface significantly enhance fiber surface roughness, especially on a nano-scale, and thus increase mechanical interlocking between CFs and matrix resin aiming to improve composite interfacial strength.

**Figure 5:**
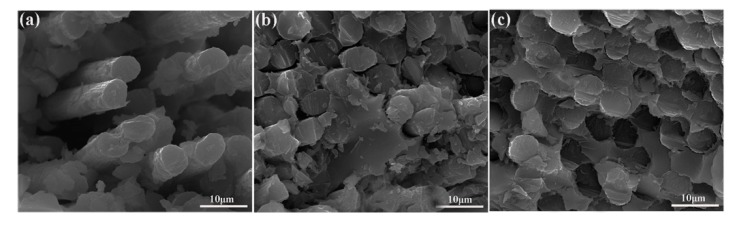
SEM morphologies of the fracture surface of MPSR composites reinforced with (**a**) untreated CF; (**b**) CF/PDA, and (**c**) CF/PDA/SiO_2_.

The above analysis of the results is also confirmed by SEM images of the composites fracture surfaces after the ILSS test, as shown in Figure 5. As for untreated CF composites (Figure 5a), there are many pulled-out fibers and holes observed on the fracture surfaces of composites, confirming the weak interfacial adhesion between untreated CF and MPSR. After the formation of PDA coating (Figure 5b), many resin fragments emerge on the fracture surfaces of CF/PDA, and simultaneously shows benign adhesion between CFs and MPSR. However, the existence of a few holes, slight breakage of CFs and a few pulled-out CFs for PDA modified composites. As for CF/PDA/SiO_2_ composites (Figure 5c), with the lack of interface debonding, fracture step, and pulled-out fibers, a favorable and desired fracture surface has been observed owing to the formation of a good PDA/SiO_2_ interface, which can increase the fiber–matrix interface combination and realize the applied load transfer effectively and high-efficient load implementation by PDA/SiO_2_ modification.

**Figure 6:**
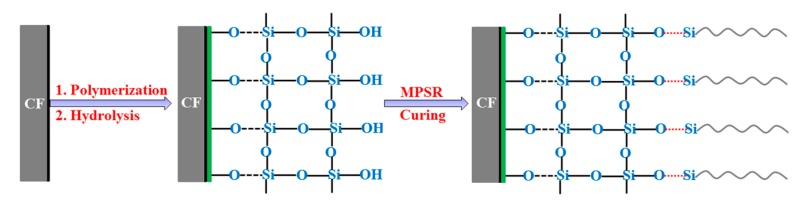
The possible mechanism of binding the SiO_2_ layer onto the PDA pre-modified CF surface and the interfacial reaction of CF/PDA/SiO_2_ composites.

Figure 6 illustrates the possible mechanism of binding the SiO_2_ layer onto the PDA pre-modified CFs surface and the interfacial reaction of CF/PDA/SiO_2_ composites. When adding CF/PDA into the solution of TEOS, the catechol groups of the PDA layer attract the hydrolyzed SiO_2_ in-situ in the solution, and the good coordination bonds can be formed between SiO_2_ nanoparticles and CF/PDA. Many key factors, like chemical bonding, mechanical interlocking, and interfacial wettability, affect interfacial adhesion between CFs and matrix. Generally, the formed chemical bonding at the interface contributes to the largest share of interface properties of composites. The presence of hydroxyl groups of CF/PDA/SiO_2_ has the high reaction activity with MPSR, creating chemical bonding at the interface of composites. The homogenous distribution and the tight binding of the SiO_2_ coating onto the fiber surface and the possible interfacial reaction may be crucial factors for better interfacial improvement.

### 3.4. Impact Property Testing of Composites

**Figure 7:**
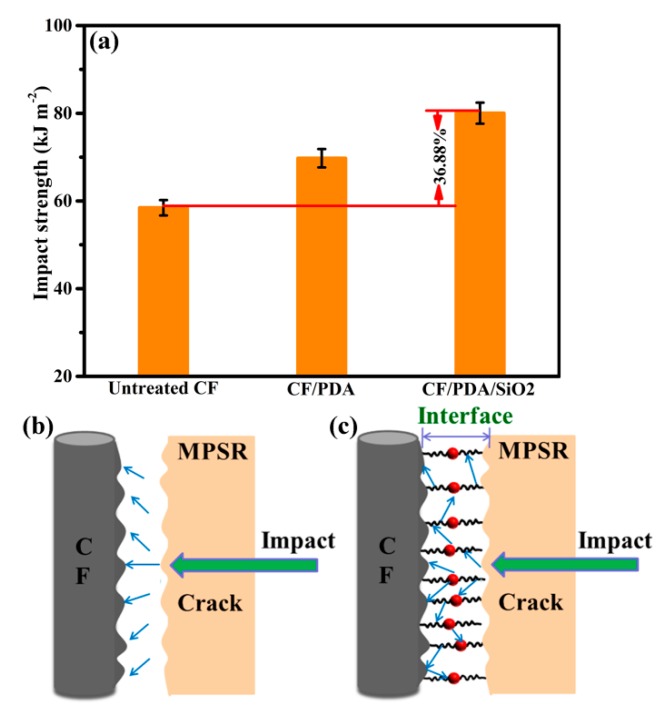
Impact testing results of composites and schematic of impact testing of composites reinforced with (**a**) untreated CF and (**b**) CF/PDA/SiO_2_.

The impact property testing results of different CFs reinforced MPSR composites are shown in Figure 7. Untreated CF composites have the low impact strength of 58.46 kJ/m^2^. After being introduced via PDA coating, impact strength of CF/PDA composites increases obviously, with the impact toughness of 69.74 kJ/m^2^ (19.30% amplification to that of untreated composites). For CF/PDA/SiO_2_ composites, the impact strength of the resulting composites enhances sharply to 80.2 kJ/m^2^, increased by 37.19% compared with that of untreated composites and 15.00% compared with that of CF/PDA composites. Such an improvement in impact toughness can be related to the formation of the PDA/SiO_2_ stratified interface, which serves as a shielding layer to prevent the crack tips from directly contacting the surface of CFs and cause the crack path to deviate away from the fiber surface to the interface region of composites. Moreover, the pull-out or rupture of tightly binding SiO_2_ nanoparticles at the interface can consume a great deal of additional energy, which contributes to the increase of the composites impact strength. The schematic illustration of the composites interface is shown in Figure 7a,b.

### 3.5. Hydrothermal Aging Resistance Testing of Composites

**Figure 8:**
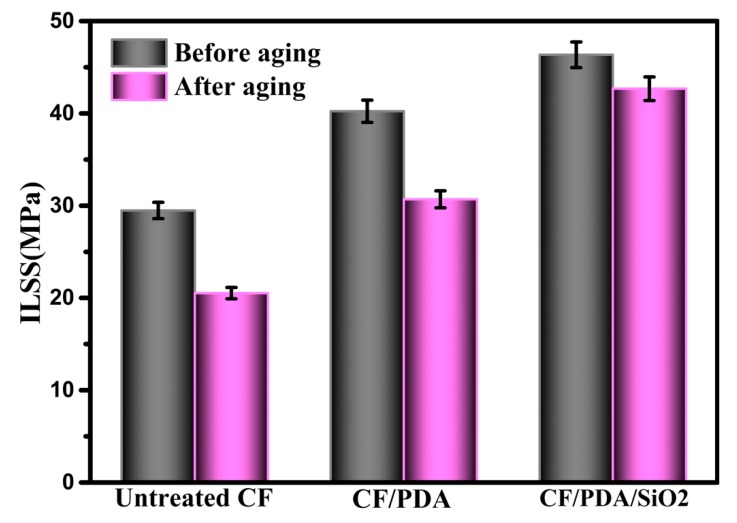
ILSS of composites before and after hydrothermal aging treatment.

Carbon fiber reinforced polymer composites as high-performance engineering materials are widely used in extreme weathering environments with high humidity and temperatures. CFs composites can absorb moisture, which severely affects their long-term durability and overall performance. Hence, higher requirements on hydrothermal aging resistance have been put forward. In the work, the modification strategy is also expected to improve their hydrothermal aging resistance. ILSS values with and without hydrothermal aging of untreated CF, CF/PDA, and CF/PDA/SiO_2_ composites are presented in Figure 8. ILSS values of untreated CF composites decrease sharply from 29.47 MPa without hydrothermal aging to 20.52 MPa after aging, with ILSS retention ratios of 69.63%. In fact, CFs virtually absorb no moisture, and absorption is largely matrix-dominated. Water uptake is mainly affected via the hydrophilic characters of matrix resin and fiber reinforcement, interfacial adhesion between the reinforcement and matrix resin, microcracks as well as voids [35]. Moisture permeation is dominated by diffusion, capillarity, and/or transport by microcracks. Based on the weak quality of the interface between untreated CF and MPSR, defects, voids as well as microcracks are easily formed under the hydrothermal aging condition, and many water molecules penetrate into the interface of composites with the aid of these microcracks or voids, leading to interfacial debonding and matrix cracking. Hence, the aging treatment deteriorates matrix resin and fiber–matrix interfaces, reducing the overall interfacial strength of the resulting composites. After 48 h of hydrothermal aging, the ILSS retention ratio of CF/PDA composites is 76.26%, which declines much slower compared to that of untreated CF composites. However, the ILSS value of CF/PDA/SiO_2_ composites after aging is 42.67 MPa with a slight decrease of 7.94%. The tested samples failed via delamination′s at the specimen mid-plane. Moisture penetration by the carbon fiber/matrix resin surface results in interfacial debonding and matrix plasticization. The formed good compaction of hierarchical PDA/SiO_2_ layers used as the buffer layers helps to inhibit the formation of microcracks and voids at the interface, and thus prevent water molecules from penetrating and damaging the interface and matrix resin effectively. Additionally, the introduced Si–O–Si bonds are to be destroyed by needing more energy and a long period of time. Hence, CF/PDA/SiO_2_ composites show the best hydrothermal aging resistance in the three types of fibers. The aging treatment has a strong effect on matrix-dominated properties due to the degradation of matrix resin and interface, resulting in a subsequent decrease in interfacial strength of composites inevitably. An ILSS retention ratio of CF/PDA/SiO_2_ composites is relatively high compared to other types of fiber’s composites, owing to the introduced hierarchical PDA/SiO_2_ layers.

## 4. Conclusions

In summary, a facile and effective strategy to modify CFs by self-polymerized polydopamine and subsequent hydrolysis of tetraethoxysilane has been reported to change interfacial microstructure and properties of MPSR composites. Characterization results indicated that SiO_2_ has been successfully deposited onto the pre-modified CFs surface. The hierarchical PDA/SiO_2_ layer led to an increased surface roughness for mechanical interlocking and surface polarity for interfacial wettability and reaction with a greater degree to that of CF/PDA as compared to untreated CF. As a result, CF/PDA/SiO_2_ composites have the highest enhancement of interfacial strength and impact toughness, affording the ILSS value (46.35 MPa), IFSS value (57.26 MPa) as well as impact toughness value (80.2 kJ/m^2^). In addition, the formation of PDA/SiO_2_ interface and the introduction of Si–O–Si bonds could effectively protect the interface from penetration of water molecules, leading to the best hydrothermal aging resistance among the three studied fibers.

## Figures and Tables

**Table 1:** Contact angles and surface energy of different CFs.

Samples	Contact Angles (°)		Surface Energy (mN·m^−1^)		
	*θ*_water_	*θ*_diiodomethane_	*γ^d^*	*γ^p^*	*γ*
Untreated CF	78.50	58.91	29.20	6.66	35.86
CF/PDA	51.36	49.72	19.74	34.43	54.17
CF/PDA/SiO_2_	44.32	43.26	22.25	37.93	60.18
